# Correlation between Infrared Absorption and Lithium Sublattice Disorder in Magnesium-Doped Lithium Niobate

**DOI:** 10.3390/ma16020797

**Published:** 2023-01-13

**Authors:** Andreas Kling

**Affiliations:** Centro de Ciências e Tecnologias Nucleares, Instituto Superior Técnico, Universidade de Lisboa, Estrada Nacional 10, km 139.7, P-2695-066 Bobadela, Portugal; akling@ctn.tecnico.ulisboa.pt

**Keywords:** lithium niobate, infrared absorption, ion beam analysis, doping, lattice structure, defect structure

## Abstract

Lithium niobate is a ferro- and piezoelectric material with excellent optical properties and a wide variety of applications. The defect structures of congruent and Mg-doped crystals are still under intense discussion. In this work, undoped lithium niobate and magnesium-doped lithium niobate grown from congruent melt with the addition of 0 to 9 mol% MgO were investigated by infrared absorption, establishing the dependence of the absorbance on the Mg-doping level in two bands related to OH${}^{-}$ stretching vibrations. The absorption band at 3485 cm${}^{-1}$ peaks at a MgO concentration in melt of 1 mol% and vanishes for MgO concentrations above the threshold level for optical damage suppression (4.8 mol%). A corresponding peak occurs in the minimum yield of the ${}^{7}$Li(p,α)${}^{4}$He reaction during ion channeling measurements, indicating a maximum of disorder in the Li sublattice. A possible explanation for this correlation is the attribution of this absorption band to ilmenite stacking fault sequences instead of isolated Nb${}_{\mathrm{Li}}$ antisites in undoped and low-doped material. On the other hand, the OH${}^{-}$ absorption band at 3535 cm${}^{-1}$ stays weak up to the MgO concentration threshold, and then increases, hinting to a defect related to the increase of vacancies due to the lack of charge compensation.

## 1. Introduction

Lithium niobate (LiNbO${}_{3}$) is a ferro- and piezoelectric material with excellent optical properties and a wide variety of photonics [1] and other applications, e.g., in spin pumping systems [2]. However, Ashkin et al. [3] observed that congruent material (i.e., crystal grown from a melt with a composition of 48.5 mol% Li${}_{2}$O and 51.5 mol% Nb${}_{2}$O${}_{5}$) suffers an alteration of the refractive index when irradiated by a 10 mW laser beam with a wavelength of λ = 515 nm focused to a spot with a radius of 0.03 mm (ca. 35 kW/cm${}^{2}$). This photorefractive effect (or “optical damage”) causes a defocusing of the light beam, which is highly detrimental to the use of lithium niobate in applications that require high light intensities, e.g., lasers, non-linear optical waveguides, second harmonic generation, optical parametric oscillators, and related applications. On the other hand, it is beneficial for holographic recording devices.

The photorefractive effect is caused by the drift of electrons out of the center of the incident light beam to a more peripheral area. The resulting electrostatic field causes the refractive index changes through the electro-optic effect inherent to the material [4]. The optical damage increases with the amount of Nb-excess in non-stoichiometric material [5], and is absent in stoichiometric LiNbO${}_{3}$ [6]. It is therefore generally accepted that Nb located in positions other than regular Nb-sites is the source of this effect. Three different models have been suggested for the type of intrinsic defects related to non-stoichiometry: lithium vacancies [7], niobium vacancies [8]—both predicting the occupation of regular Li-sites by Nb (anti-site Nb)—and the occurrence of stacking faults [9] with an ilmenite-type structure. For a detailed discussion of the models see, e.g., [10].

There are two principal possibilities for overcoming the undesired photorefracctive effect: (1) the use of flux-grown stoichiometric lithium niobate [11] which has no Nb-excess and (2) doping with metal ions that replace the excess Nb without causing a photorefractive effect on their own. The technologically most important dopant is Mg, that suppresses the optical damage if more than 4.8 mol% MgO are added to the congruent melt [12].

While studies on the optical properties of doped Mg are innumerable, attempts to determine the lattice location of Mg—and consequently the mechanism to replace the Nb excess mechanism—by experimental means have been scarce. A first NMR study by Feng et al. [13] on Mg-nuclei failed to detect any change at the threshold concentration, while Yatsenko et al. [14] concluded indirectly from the signals of ${}^{7}$Li and ${}^{93}$Nb that Mg should be located solely on Li-sites below and at Li- and Nb-sites above the threshold. An ion beam channeling investigation [15] concluded that Mg occupies a lattice site at the center of the Li-octahedron for MgO-concentration < 1.5 mol% and regular Li- and Nb-octahedron-center sites above this value; no change in the lattice occupation mechanism was observed at the threshold value.

Hydrogen in lithium niobate is—due to its technological importance in optical waveguide formation—another important issue in LiNbO${}_{3}$ and has therefore been studied intensively (a comprehensive review is given in [16]). Infrared absorption induced by defect-related OH${}^{-}$ vibrational oscillations, caused by ubiquitous hydrogen impurities, has been reported for the first time by Smith et al. [17] with an absorption band showing up at 3500 cm${}^{-1}$ (2.86 μm) in congruent LiNbO${}_{3}$. The first detailed IR-absorption study on congruent Mg-doped LiNbO${}_{3}$ [18] confirmed the existence of a 2.87 μm (3484 cm${}^{-1}$) band in crystals doped with up to 4.5 mol% MgO. In addition, a new absorption band at 2.83 μm (3540 cm${}^{-1}$) was found to arise for doping concentrations of 4.5 mol% and above. Further investigations on samples grown from congruent material by Jin et al. [19] showed an absorption band at 3470 cm${}^{-1}$ (2.88 μm) for concentrations up to 5.0 mol% MgO, which was fully substituted by a 3534 cm${}^{-1}$ (2.83 μm) band for 7.5 and 10 mol% MgO. Kong et al. [20] report the OH${}^{-}$ absorption band occurring at 3483 cm${}^{-1}$ (2.87 μm) in a sample with 4 mol% MgO. The sub-threshold Mg-concentration range was studied by Kovacs et al. [21], focussing on the influence of the Li/Nb ratio on the infrared absorption of crystals grown with 1 to 5.4 mol% of MgO. In crystals grown from Li-rich ([Li]/[Nb] = 1.1 to 1.2) melts, the absorption at 2.83 μm was already observed for a MgO concentration of 2.7 mol%. A more recent study [22,23] reports three-phase behavior for samples grown from stoichiometric melt with a MgO concentration ranging from 0.19 to 5.91 mol%: (i) for [MgO] = 0.19 to 3.02 mol% absorption bands at 3466 cm${}^{-1}$ (2.89 μm) and 3486 cm${}^{-1}$ (2.87μm); (ii) for [MgO] = 5.29 mol% at 3535 cm${}^{-1}$, and (iii) at 5.91 mol% at 3483 cm${}^{-1}$ (2.83 μm), i.e., close to the value for low-doped material.

The first study on Mg-doped near-stoichiometric LiNbO${}_{3}$, presented by Furukawa et al. [24], showed a complete absence of the 2.88 μm band in all Mg-doped crystals. An absorption band at 3466 cm${}^{-1}$ (2.89 μm), corresponding to that of undoped near-stoichiometric LiNbO${}_{3}$ [11,24], was reported for one of the samples with 1 mol% MgO, while for higher concentration only the band at 3534 cm${}^{-1}$ (2.83 μm) has been observed. The latest study on IR-absorption [25] also investigates near-stoichiometric samples grown using a very high [Li]/[Nb] ratio (1.38) or produced using the vapor transport equilibrium (VTE) technique for MgO melt concentrations of 0, 0.5 and 1 mol%. In the first type of sample, clear absorption peaks were observed at 3466 cm${}^{-1}$ (2.89 μm) and 3480 cm${}^{-1}$ (2.87 μm) for 0.5 mol%, while for 1 mol% only one band at 3535 cm${}^{-1}$ (2.83 μm) was detected. Mg-doped VTE samples show all three bands, but with very low intensity.

Ion channeling is an important method for the study of lattice defects and dopant lattice location determination and has been intensively applied to the case of LiNbO${}_{3}$ (for an extensive review and details on the technique see [26]). While the Rutherford Backscattering Spectroscopy under channeling conditions is the most widely applied method, the investigation of light elements relies on Nuclear Reaction Analysis (NRA). In the case of lithium niobate, the usefulness of the ${}^{7}$Li(p,α)${}^{4}$He reaction for the study of the lithium sublattice in lithium niobate has been demonstrated in various previous studies [15,27,28,29]. Its application on Mg-doped crystals indicated that a maximum of Li sub-lattice disorder occurs in the range of 0.5 to 1 mol% of MgO for all axial directions except for the 〈0001〉-axis [28]. The latter indicates that all cationic positions (host and dopant) are fully aligned with the c-axis. The interpretation of the data for the remaining axial directions pointed at a weakening of the ion repelling continuum potential produced by the aligned Li-atoms due to the substitution of Nb${}_{\mathrm{Li}}$ (Z = 41) by Mg${}_{\mathrm{Li}}$ (Z = 12). Further, the observation that Mg occupies sites in the center of the Li-octahedron instead of the regular Li-site [15] in this concentration range enhances the disturbance of the ion channeling effect. The significantly stronger NRA channeling effect in near-stoichiometric compared to congruent LiNbO${}_{3}$ [30,31] means that the disturbance by disorder in the Li-sublattice outweighs the potential enhancement due to Nb${}_{\mathrm{Li}}$.

The present paper intends to combine the results from IR-absorption measurements and ion channeling experiments probing the Li-sublattice of undoped and Mg-doped lithium niobate using a set of crystals grown under identical conditions to achieve a consistent model of the cation defect structure of these materials.

## 2. Experimental Details

The crystals investigated were grown by the Czochralski method from a congruent melt ([Li]/[Nb] = 0.94), to which MgO was added in a concentration range from 0.5 to 9.0 mol%. For a detailed description of the growth method, refer to [32]. The distribution coefficient for magnesium was determined to be 1.2 [33]. The crystals were investigated in depth with regard to their ferroelectric, piezoelectric and optical properties, and lattice site location of Mg [15,34,35]. In the following, the MgO concentration values indicated always refer to melt compositions.

The infrared absorption studies were performed using a CARY 5G spectrophotometer. Measurements were carried out with unpolarized light from a tungsten lamp in transmission geometry. The thickness of all samples investigated was 0.5 mm.

New ion beam measurements for this work were performed at the 2.5 MV Van-de-Graaff accelerator facility of the Instituto Superior Técnico. In order to be compatible with previous measurements presented in [28,30,31], a 1.75 MeV proton beam was used to study the ${}^{7}$Li(p,α)${}^{4}$He reaction under channeling conditions for the 〈$02\overset{¯}{2}1$〉 axial direction of the LiNbO${}_{3}$ crystals. Backscattered protons and alpha particles arising from the nuclear reaction were detected simultaneously, using silicon surface barrier detectors located at an angle of 165${}^{\circ }$ with respect to the beam. The depth interval investigated for obtaining the minimum yield for the nuclear reaction was 1 μm, as in the studies mentioned above.

## 3. Results and Discussion

**Figure 1:**
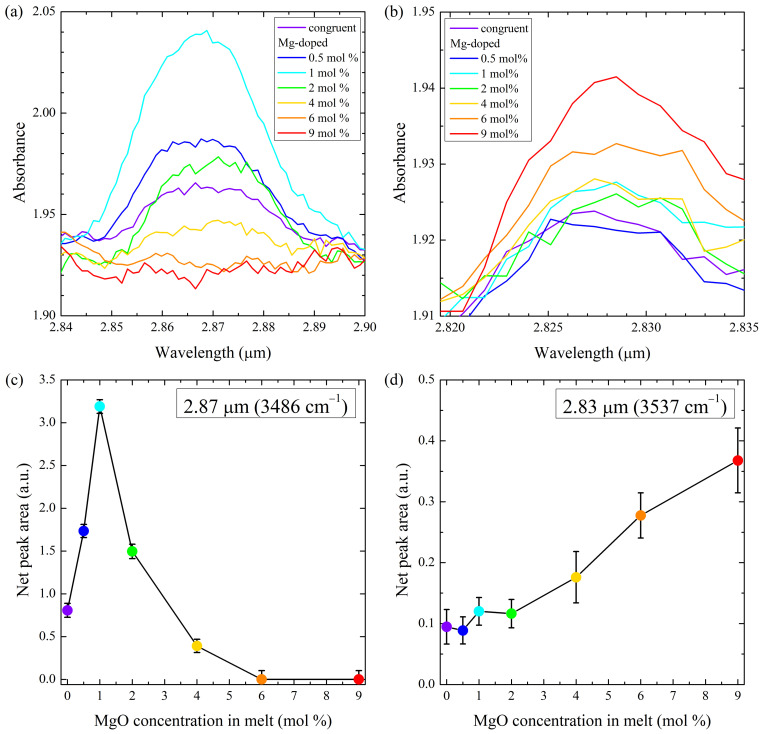
(**a**,**b**) Infrared absorption bands at 2.87 μm and 2.83 μm for congruent and Mg-doped lithium niobate samples. (**c**,**d**) Areas under the respective absorption peaks in dependence of the MgO concentration in the crystals. The symbol colors correspond to the line colors used in the upper part.

Figure 1a,b show the infrared absorption spectra for congruent and Mg-doped lithium niobate crystals in two bands—2.87 μm (3485 cm${}^{-1}$) and 2.83 μm (3535 cm${}^{-1}$). The net area below the absorption peaks was calculated, fitting Gaussian curves to the data in the ranges from 2.840 μm to 2.900 μm and from 2.815 μm to 2.835 μm, respectively, and was plotted in Figure 1c,d. The two absorption bands show distinct dependencies on the Mg-concentration.

The absorption band at 2.87 μm has been generally attributed to the existence of Nb${}_{\mathrm{Li}}$ in congruent, i.e., lithium deficient LiNbO${}_{3}$, and is found to be absent in flux-grown stoichiometric material [11,24], which is supposed to have a perfectly ordered lattice. The strong increase in this absorption band at low MgO concentrations observed would therefore imply an increase in Nb antisites in this concentration range. However, all models on Mg-incorporation published so far defend the idea that Mg should replace Nb${}_{\mathrm{Li}}$ continuously with increasing Mg-concentration and eliminate this defect completely for concentrations above the optical damage threshold [34,36,37,38,39,40] or at 1.5 mol% [41].

**Figure 2:**
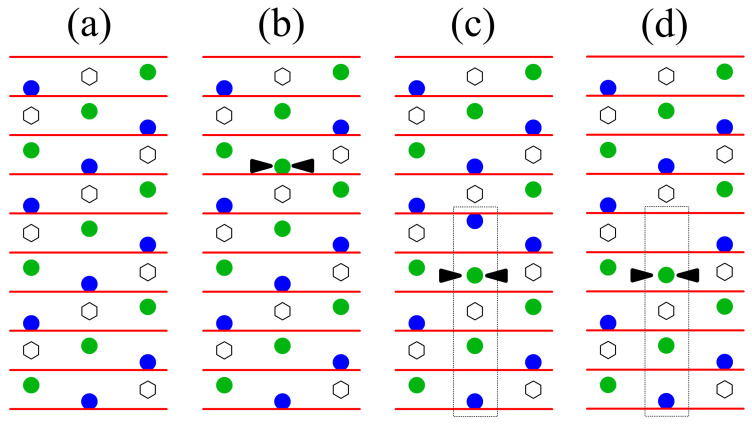
Stacking models for the intrinsic defect structure of LiNbO${}_{3}$: (**a**) regular stacking, (**b**) Nb-antisites (extra Nb indicated by arrows), (**c**) ilmenite stacking faults (boxed area) and (**d**) as (**c**) but with Li vacancy (acts like a Nb-antisite). Nb is represented by green, Li by blue and free octahedral positions by white symbols, and the oxygen layers are depicted as red lines (from [26]).

A possible solution to this apparent contradiction with the IR absorption results is to attribute the observed absorption band to stacking fault sequences with an ilmenite structure in the material (Figure 2). In this case, Nb-antisites can be reinterpreted as ilmenite-type stacking faults in which the Li-site is vacant [10], as depicted in Figure 2d, in the following referred to as Nb${}^{\mathrm{ilm}}$. Additionally, complete stacking faults (Figure 2c) could exist in the congruent material. A further increase of their concentration during Mg-doping can be achieved without altering the material’s stoichiometry. Furthermore, stoichiometric LiNbO${}_{3}$ produced by VTE using Li-indiffusion shows—in contrast to flux-grown stoichiometric crystals—a residual absorption band at 2.88 μm [25,42]. It seems unlikely that isolated Nb${}_{\mathrm{Li}}$ should not be completely removed by this process. which takes place at about 1100 ${}^{\circ }$C [43]. On the other hand, complete stacking faults—pre-existing or formed by Li-incorporation in “defective” ones—may remain stable as soon as stoichiometry is achieved; in the absence of vacancies, the dissolution of such a stacking fault would require the simultaneous swapping of neighboring Nb${}^{\mathrm{ilm}}$ and Li${}^{\mathrm{ilm}}$.

The ion channeling study on the lattice site location of Mg in this set of crystals mentioned above [15] indicated different incorporation mechanisms for MgO-concentrations up to 1 mol% (center of the Li octahedron). Eventually, the more symmetric position of Mg (in comparison to the regular Li site) in the crystal lattice favors the formation of—in contrast to LiNbO${}_{3}$—inversion-symmetric stacking faults in its vicinity.

With a further increase in MgO concentration, the absorption band becomes weaker again and is completely suppressed for Mg-concentrations above 4 mol% MgO. The observation corroborates the connection of the complete extinction of this lattice defect with the suppression of the optical damage phenomenon. This observation is in accordance with previous studies on crystals grown from congruent melts [17,18,19]. The only exception is [21], which reports the absorption band to persist at 5.2 mol% MgO. On the other hand, the latter study sees a full suppression of the 2.87 μm band for lower Mg-concentrations (4 mol% and 2.7 mol% MgO with [Li]/[Nb] ratios of 1.1 and 1.2, respectively) in crystals grown from Li-rich melts. Li-excess in the melt is known to reduce, but not to eliminate, the Nb-excess in the crystals [32,33]. Therefore, lower Mg-concentrations are sufficient to expel the remaining Nb located in the Li-octahedron. The results of [22,23] also show an extinction of the 2.87 μm band at 5.29 mol%. On the other hand, a reappearance of the absorption band at 5.91 mol% MgO was found and attributed to a threshold for the incorporation of Mg occurring at 5.5 mol% MgO. However, the results of the present study and the work of Jin et al. [19] agree that this absorption band is absent for MgO-concentrations between 6 and 10 mol%.

The 2.83 μm absorption band is much weaker and shows an almost constant value up to 4 mol% MgO, and then increases. The occurrence of this absorption band, attributed to Mg occupying regular Nb sites [20], has not been reported in the two other studies for MgO concentrations below the optical threshold. In [22], its absence is visible in the spectra, while [19] and [21] do not depict this region of the IR-absorption spectra for low Mg concentrations. However, the proposed defect type, Mg${}_{\mathrm{Nb}}$, cannot exist in undoped material and, therefore, in this case an alternative explanation might be necessary. The low values of absorption indicate a quite low concentration of this defect. For the congruent material, a tentative interpretation could be the existence of a residual amount of Nb vacancies introduced during the growth process. One also should bear in mind that the crystal growth methods used by each research group show slight differences in the conditions used, which may influence the properties of the resulting material in a subtle manner.

Also, it is difficult to understand that the amount of this defect type increases for concentrations above 4 mol%. Studies on the Mg-incorporation [15,36,41] favor the incorporation of Mg on Li and Nb sites at ratios [Mg${}_{\mathrm{Li}}$]:[Mg${}_{\mathrm{Nb}}$] of 1:1 and/or 2:1 in this concentration range, which leads only to the formation of additional Li-vacancies, or of 3:1, which would be charge compensating [36]. One could speculate that Mg-dimers on neighboring sites (Mg${}_{\mathrm{Li}}$-Mg${}_{\mathrm{Nb}}$) [38] could create locally oxygen deficient surroundings, i.e., with the stoichiometry of MgO. This could force the formation of Nb- and/or Li-vacancies to compensate the oxygen deficiency. Mg-dimers are also considered in [22] to be the source of the 2.83 μm absorption band, in this case by forming Mg${}_{\mathrm{Li}}$-Mg${}_{\mathrm{Nb}}$-OH complexes. Although no experimental evidence for the formation of these dimers exists, the striking similarity of the oxygen sublattices of MgO in the (0001)-plane and LiNbO${}_{3}$ in the (111)-plane [44] may serve as an indication that the formation of such small defect clusters with MgO configuration could take place.

**Figure 3:**
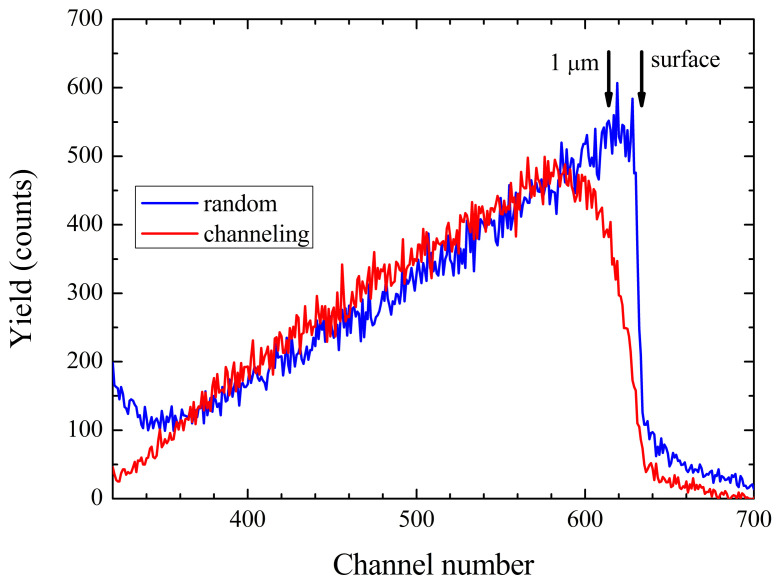
The spectra of the α-particles emitted in the ${}^{7}$Li(p,α)${}^{4}$He reaction for aligned (〈$02\overset{¯}{2}1$〉-axis) and random incidence of the protons. The spectra are analyzed in the range of 0 (surface) to 1 μm depth.

Figure 3 shows an aligned (〈021〉-axis) and a random incidence spectrum of the alpha particles produced in the ${}^{7}$Li(p,α)${}^{4}$He reaction to illustrate the effect of channeling. The minimum yields ($\chi_{min}$), i. e., the ratio of counts in aligned and random incidence cases, are calculated in this study for a depth of 1 μm corresponding to the alpha particle energy range of 7.357 to 7.566 MeV. In lithium niobate, the guiding effect for ions is mainly established by the repelling Coulomb potential of the Nb (Z = 41) atomic chains in all axial directions [26]. This leaves the chains consisting of Li atoms (Z = 3)—and to a lesser extent those of O (Z = 8)—very sensitive to disturbances in their sub-lattices, which manifest themselves in an increase of the minimum yield with respect to a perfect lattice.

**Figure 4:**
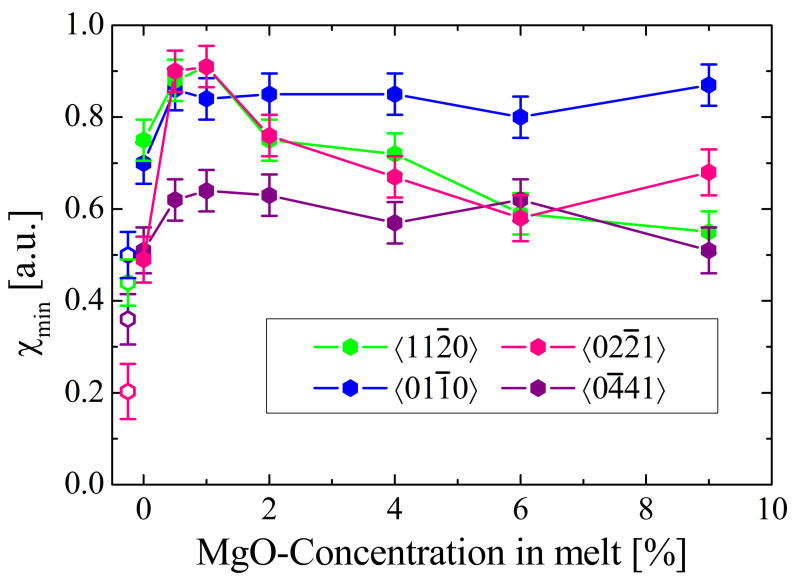
Dependence of the minimum yield for the ${}^{7}$Li(p,α)${}^{4}$He reaction in various axial directions in dependence of the MgO concentration (full symbols). Values for 〈$11\overset{¯}{2}0$〉, 〈$01\overset{¯}{1}0$〉 and 〈$0\overset{¯}{4}41$〉 are taken from [28]; values for 〈$02\overset{¯}{2}1$〉 from this work. The open symbols represent values for undoped near-stoichiometric material taken from [30,31] and are slightly shifted to the left for better visibility.

The dependence of the minimum yields for the congruent and MgO-doped samples for the 〈$02\overset{¯}{2}1$〉-direction (this work) is depicted in Figure 4, together with data for other important axial directions for the same samples retrieved from a previous work [28] and for near-stoichiometric material [30,31]. For all axial directions, an increase of $\chi_{min}$ is observed for low MgO concentrations (≤1 mol%) with respect to the congruent material. For the 〈$02\overset{¯}{2}1$〉 and the 〈$11\overset{¯}{2}0$〉, $\chi_{min}$ actually peaks for these concentrations—as the IR-absorption at 2.87 μm—and then falls to values comparable to the congruent case.

**Figure 5:**
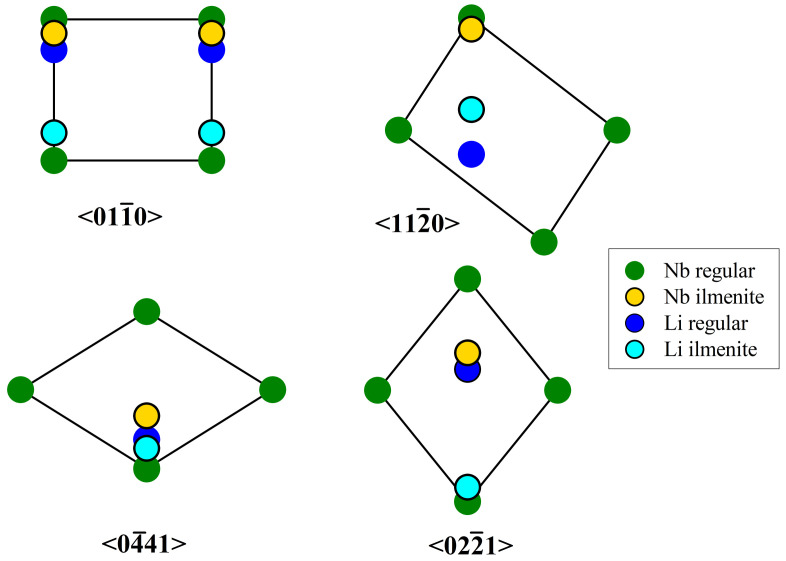
Channel projections with regular and ilmenite type lattice sites for Li and Nb for the four crystallographic directions studied (adapted from [26]).

The $\chi_{min}$ values for near-stoichiometric material, which is supposed to have almost perfectly ordered Li- and Nb-sublattices (i.e., free of any Nb occupying positions in the Li octahedron) [30], can serve as a reference. The comparison with the values for the congruent crystal demonstrates the sensitivity of the method to disorder in the Li-sublattice of lithium niobate. If excess Nb in congruent lithium niobate occupies regular lithium sites (Nb${}_{\mathrm{Li}}$), the dominating effect from the viewpoint of channeling should be an enhancement of the repelling potential and, consequently, should lead to a decrease of $\chi_{min}$ with respect to the near-stoichiometric case—which contradicts the observation. On the other hand, ilmenite stacking faults would—due to symmetry requirements—place Nb atoms slightly outside their respective atomic chains, leading to dechanneling. Although Nb${}^{\mathrm{ilm}}$ acts in most aspects like Nb${}_{\mathrm{Li}}$, in terms of channeling it exhibits very distinct behavior. The different location occupied in the channel results in an enhancement of nuclear reaction probability with lithium, and in an increase of $\chi_{min}$—in agreement with the observation. The highest impact is expected to be observed for the 〈$02\overset{¯}{2}1$〉 axial direction, since Nb${}^{\mathrm{ilm}}$ is located in the center of the channel (see Figure 5) where the ion flux is maximum. Heavy atoms located in such a flux peak strongly enhance the scattering of the ions, reducing the number of ions in channeling mode. These dechanneled ions increase the nuclear reaction probability with the Li atoms in the channel. The effect is especially strong for the 〈$02\overset{¯}{2}1$〉-axis, since the regular Li-site is found in a near-central position, resulting in the dramatic increase of $\chi_{min}$.

For the other axial directions the strong increase of $\chi_{min}$ observed for low MgO concentrations cannot be solely understood by the dechanneling induced by Nb${}^{\mathrm{ilm}}$ and the incorporation of Mg in the Li-octahedron. Again, the interpretation given for the increase in the 2.87 μm absorption (formation of “complete” ilmenite stacking faults) can resolve this question. These stacking faults contain as an additional defect Li${}^{\mathrm{ilm}}$, displaced from the regular Li-site. An axial direction in which this defect is in a location near the channel center is 〈$12\overset{¯}{2}0$〉 (Figure 5) which, indeed, shows strong peaking for low Mg-concentrations. For the other two axial directions (also depicted in Figure 5), Li${}^{\mathrm{ilm}}$ and Nb${}^{\mathrm{ilm}}$ are located far more peripherally, reducing their influence but still leading to a noticeable increase of $\chi_{min}$.

For higher Mg-concentrations, various effects will influence the evolution of the lithium nuclear reaction minimum yield exerting opposing effects: (1) the reduction of ilmenite stacking faults will tend to reduce the dechanneling and lower $\chi_{min}$; (2) the incorporation of Mg on regular Li sites will enhance the repelling effect of the Li-strings and also lower the $\chi_{min}$; (3) the incorporation of Mg on sites in the center of the Nb-octahedron (as suggested by [15]) increases the dechanneling and $\chi_{min}$; while (4) the incorporation of Mg on regular Nb sites would have no noticeable effect. The dominating effect seems to be (1), explaining the decrease in the minimum yield above 1 mol% MgO well, especially for the 〈$11\overset{¯}{2}0$〉 and 〈$02\overset{¯}{2}1$〉 directions. Again, this is in accordance with the decrease observed in the IR-absorption, as expected for a gradual extinction of the stacking faults.

## 4. Conclusions

Comparing the results from two very distinct methods of research (IR-absorption and nuclear reaction probabilities in channeling conditions) showed interesting correlations. The peaking effect in the 2.87 μm IR-absorption and the lithium nuclear reaction minimum yield occur in the same Mg concentration range. A consistent explanation can be given, assuming that ilmenite stacking faults are present in congruent and increase in number in low-level MgO-doped crystals. This can be seen as a further hint to the existence of this defect type in non-stoichiometric lithium niobate. The outcome of the study also corroborates that the elimination of the defects related to the 2.87 μm absorption band goes in line with the suppression of the optical damage effect in this material. Evidence has also been found that the 2.83 μm absorption band may need a new interpretation in terms of the defect associated with it, although no positive affirmation of its type can be presented at this point.

## Data Availability

The data presented in this study are available on request from the corresponding author.
